# Multiple-Octave-Spanning Vibration Sensing Based on Simultaneous Vector Demodulation of 499 Fizeau Interference Signals from Identical Ultra-Weak Fiber Bragg Gratings Over 2.5 km

**DOI:** 10.3390/s18010210

**Published:** 2018-01-12

**Authors:** Yi Li, Li Qian, Ciming Zhou, Dian Fan, Qiannan Xu, Yandong Pang, Xi Chen, Jianguan Tang

**Affiliations:** 1 National Engineering Laboratory for Fiber Optic Sensing Technology, Wuhan University of Technology, Wuhan 430070, China; liyi590lucky@163.com (Y.L.); l.qian@utoronto.ca (L.Q.); fandian@whut.edu.cn (D.F.); xqn_990822@163.com (Q.X.); pangyandong1000@163.com (Y.P.); skyqnet@163.com (X.C.); tangjg@whut.edu.cn (J.T.); 2 School of Science, Wuhan University of Technology, Wuhan 430070, China; 3 Department of Electrical and Computer Engineering, University of Toronto, Toronto, ON M5S 1A1, Canada; 4 Key Laboratory of Fiber Optic Sensing Technology and Information Processing, Ministry of Education, Wuhan University of Technology, Wuhan 430070, China

**Keywords:** fiber-optic sensors, fiber Bragg gratings, vibration sensing, low-frequency range

## Abstract

Multi-point vibration sensing at the low frequency range of 0.5–100 Hz is of vital importance for applications such as seismic monitoring and underwater acoustic imaging. Location-resolved multi-point sensing using a single fiber and a single demodulation system can greatly reduce system deployment and maintenance costs. We propose and demonstrate the demodulation of a fiber-optic system consisting of 500 identical ultra-weak Fiber Bragg gratings (uwFBGs), capable of measuring the amplitude, frequency and phase of acoustic signals from 499 sensing fibers covering a total range of 2.5 km. For demonstration purposes, we arbitrarily chose six consecutive sensors and studied their performance in detail. Using a passive demodulation method, we interrogated the six sensors simultaneously, and achieved a high signal-to-noise ratio of 22.1 dB, excellent linearity, phase sensitivity of around 0.024 rad/Pa, and a dynamic range of about 38 dB. We demonstrated a frequency response flatness of <1.2 dB in the range of 0.5–100 Hz. Compared to the prior state-of-the-art demonstration using a similar method, we have increased the sensing range from 1 km to 2.5 km, and increased the frequency range from 0.4 octaves to 7.6 octaves, in addition to achieving sensing in the very challenging low-frequency range of 0.5–100 Hz.

## 1. Introduction

Fiber vibration sensors have been increasingly recognized as a promising technology for vibration sensing, such as seismic sensing [1] and underwater acoustic imaging [2]. At present, the most common methods for vibration sensing are various interferometers including Sagnac interferometers [3], Mach–Zehnder interferometers [4], Michelson interferometers [5] and composite interferometers [6]. The main advantages of these interference structures are their high sensitivity and wide detectable frequency range, achieved by detecting the optical phase changes caused by vibrations [7]. However, generally, these interference structures are difficult for large-scale multiplexing or location-resolved distributed sensing. Phase sensitive optical time domain reflectometers (φ-OTDRs), on the other hand, show a significant advantage with regard to distributed vibration detection. φ-OTDR utilizes a narrow line width laser source to form inference between nearby Rayleigh backscattered light waves [8,9]. However, due to the weak Rayleigh backscattering along the fiber, and the fact that the backscattered light at certain positions of the fiber is not always stable, the signal-to-noise ratio (SNR) of the demodulated φ-OTDR signal is low. Improved SNR can be obtained by performing data averaging [10], but this reduces the speed of detection significantly. In order to increase the signal level, the combination of fiber Bragg gratings (FBGs) and φ-OTDR has been proposed, where the reflections come from the FBGs instead of Rayleigh backscattering, resulting in orders-of-magnitude higher signal levels [11]. In such a system, the detected signal is typically Fizeau interference formed by reflections from two adjacent FBGs [12]. A large-scale multiplexed vibration sensing system has been demonstrated using ultra-weak FBGs (uwFBGs) [12]. However, the frequency detection range in [12] is quite limited, from 450 Hz to 600 Hz, or 0.4 octave. Though another experiment using Rayleigh backscattering φ-OTDR has reported a frequency detection range of 50–500 Hz (or 3.3 octaves) [13], it does not involve a large array of sensing elements and has a limited sensing range of 10 m. So far, none has demonstrated the combination of multipoint sensing over a large sensing range and broadband detection in the challenging low frequency range of 0.5–100 Hz, which is of great significance to underwater acoustic imaging and detection [2].

In this paper, we demonstrate the demodulation of a fiber sensing system consisting of 499 vibration sensors, made up of 500 identical uwFBGs with 5 m of sensing fiber between adjacent uwFBGs. The system is capable of measuring acoustic signals covering a total range of 2.5 km. To demonstrate multipoint capability, we arbitrarily choose six consecutive sensors and study their performance in detail by cementing them on the inner and outer circles of a fan-shaped particleboard and driving them with sinusoidal vibration signals from a piezoelectric transducer (PZT) wafer place at the center of the circle. Our results show that the system can simultaneously demodulate the amplitude, frequency and phase of vibration signals over an unprecedented 7.6 octaves of frequency range, from 0.5 to 100 Hz, more than an order of magnitude higher, in terms of octaves, than in [12]. We note that it is particularly challenging to achieve a good signal-to-noise ratio, especially at the very low frequencies, due to 1/f noise. Thus, we believe that this system is very promising for applications requiring low frequency acoustic detections, such as seismic monitoring and submarine acoustic imaging.

## 2. Principle

**Figure 1:**
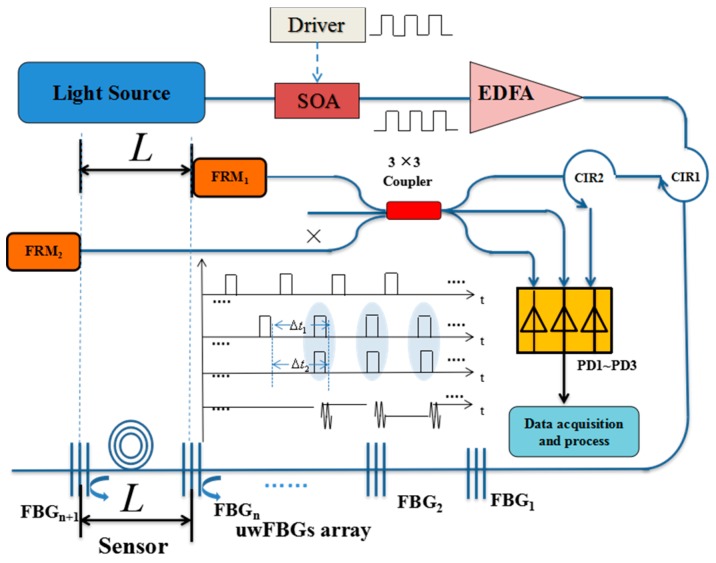
Schematic diagram of our vibration sensing system based on Fizeau interferences of identical ultra-weak fiber Bragg gratings. SOA: semiconductor optical amplifier; FRM: Faraday rotation mirror; PD1~3: high-sensitive photodetectors; DAQ: Data Acquisition Board.

Figure 1 shows the schematic configuration of our large-scale multiplexed acoustic sensing system. The amplified light pulses are injected into the uwFBG array through an optical circulator (CIR1). Five hundred identical uwFBGs (with reflectivity in the range of −30 to −40 dB) are written into the fiber online using the fiber draw process [14] at an interval of L (5 m). Every pair of adjacent uwFBGs, together with the 5 m fiber in between, form a Fizeau interferometer, which is used to sense vibration in that section. Therefore, there are altogether 499 quasi-distributed sensors in our system. (A Fizeau interferometer is basically a common-path interferometer consisting of two weak reflectors. Vibration causes an optical-path-length change of the fiber due to photoelasticity, and results in a change in the interference pattern.) The round-trip delay between the reflected pulses from any two adjacent uwFBGs is $\Delta t_{1}$. The reflected pulses then pass directly through another optical circulator (CIR2) and enter into an unbalanced Michelson interferometer employing a symmetric 3 × 3 coupler. The arm imbalance of the interferometer $\Delta t_{2}$ can be made to compensate the round-trip delay $\Delta t_{1}$ such that the reflected pulses from two adjacent uwFBGs are brought together in time to interfere (see the timing diagram shown in the insert of Figure 1). The two unbalanced arms are terminated with Faraday rotation mirrors (FRMs) to compensate for the different polarization rotations experienced by the light pulses travelling in the two arms of the interferometer.

The demodulation algorithm of the interference signal generated by the symmetric 3 × 3 coupler is well known [15]. The key advantage of using a 3 × 3 coupler based demodulator is that it does not require any active carrier modulation to extract the phase information presented by the interferometer, thus avoiding phase noise in the interferometer due to the frequency jitter of the carrier [15].

Three photodiodes (PDs) were connected at the three output ports of the 3 × 3 coupler. A total of 120 phase shifts between the signals were received by any two PDs. Accordingly, the outputs of the three PDs can be expressed as [12]:

(1) $$I_{k}=D_{k}+E_{k}cos\left[\varphi \left(t\right)+\left(k-1\right)\frac{2\pi }{3}\right]\left(k=1,2,3\right)$$

where k is the optical path index number; $D_{k}$ represents the average of the output light intensity; $E_{k}$ is the peak intensity of the interference fringes. After applying a standard demodulation algorithm [15], we can directly obtain the phase angle φ(t) in Equation (1) as a function of time: $\varphi \left(t\right)=\phi \left(t\right)+\phi_{n}\left(t\right)+\phi_{0},\;\mathrm{where}\;\phi \left(t\right),\phi_{n}\left(t\right),\phi_{0}$ are respectively the signal to be detected, the noise and the initial phase of the system. When vibration pressure affects the sensing fiber, due to the photoelastic effect, the refractive index of the fiber will change, which will change φ(t). Through the 3 × 3 demodulation algorithm, the phase, amplitude and frequency of φ(t) can be directly obtained at the same time. Therefore, it is termed simultaneous vector demodulation.

## 3. Experiments Results and Discussion

In our experiment, the light source was a distributed-feedback laser diode with a narrow line width of 150 kHz at a center wavelength of 1549.900 nm, which was matched with the center wavelength of the uwFBGs. The CW light was modulated and amplified by a semiconductor optical amplifier (SOA), which was driven by a pulsed electrical signal, and the output of the SOA became a train of 20 ns pulses with a repetition rate of 5 kHz. The pulses reflected by the uwFBGs were spaced by ~50 ns, and therefore were well separated temporally. The repetition rate was set to 5 kHz to ensure the reflected pulses did not overlap in time with the later pulses sent from the source. The optical pulses were amplified by an Erbium Doped Fiber Amplifier (EDFA) with a gain of 22.1 dB to improve the signal-to-noise ratio (SNR). The uwFBG array was fabricated during fiber draw [14], which includes 500 identical uwFBGs spaced uniformly at 5.0 m intervals, and the grating reflectivities were in the range of –30 dB to –40 dB, centered at 1549.900 nm with a 3 dB bandwidth of 1.6 nm. The reflected light pulses were separated in time by about 50 ns (10 m), and then were brought back to interfere at the 3 × 3 coupler by the unbalanced Michelson interferometer. The arm imbalance of the Michelson interferometer was 5.0 m, matched to the spacing of the uwFBGs, such that only adjacent reflected pulses interfered. Faraday mirrors (FMs) were used as reflectors of the Michelson interferometer to undo the polarization transformations of the two arms. Note that although the polarization variation due to the different arms of the interferometer was eliminated by the use of the FMs, the polarization states of the two interfering signals could still be different, due to the fact that they were reflected by different uwFBGs separated by 5 m. We also remark that even though the intensity of the interference signal was affected by the polarization states of the two interfering light pulses, the phase φ(t) was unaffected. The interference signals at the three output ports of the symmetric 3 × 3 coupler were converted into an electrical signal by three PDs (PD1–PD3), and collected by a high-speed data acquisition card NIPXI-1071 (National Instruments Co., Austin, TX, USA) with the maximum real-time sampling rate of 250 MS/s, and then uploaded to a computer for data processing and real-time display.

**Figure 2:**
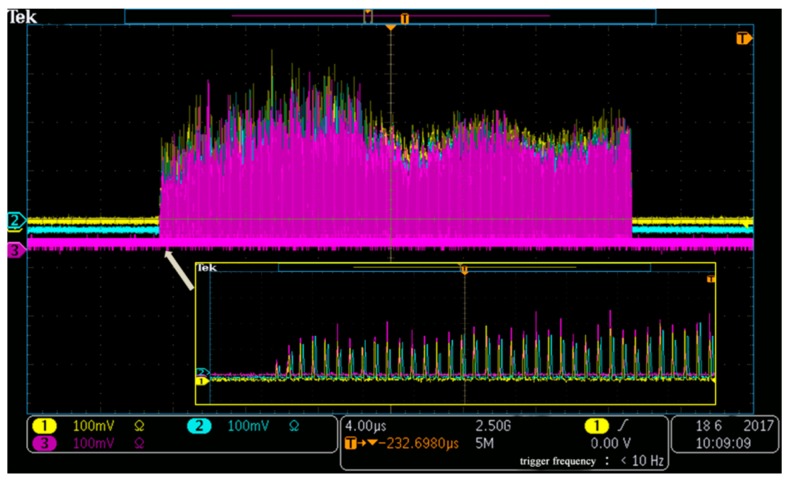
The interference signals of the 500 ultra-weak Fiber Bragg gratings (uwFBGs) obtained by three photodetectors shown on the oscilloscope. The inset shows an enlarged view of the first few pulses.

During the experiment, a piezoelectric transducer (PZT) was used as a vibration source. It was placed on a particle board with a dimension of 630 mm × 480 mm × 10 mm, on which six randomly-selected consecutive uwFGBs were placed. The PZT was driven by a sinusoidal signal produced by a signal generator (DG5251) with an amplitude of 1 V. The signals collected by NIPXI-1071 card corresponding to all 499 interference pulses are clearly visible in Figure 2, where the red, blue, and yellow lines represent the three signals detected by three PDs respectively. Each peak corresponds to a Fizeau interference signal between reflected pulses from adjacent uwFBGs in the sensor array. The intensity variation in these signals is mainly due to (a) the variation in reflectivity of the uwFBGs; (b) the variation in polarization states between the interfering pulses. The enlarged view is shown in the insert, where we can see the differences in phase and power among the three outputs of the 3 × 3 coupler more clearly.

**Figure 3:**
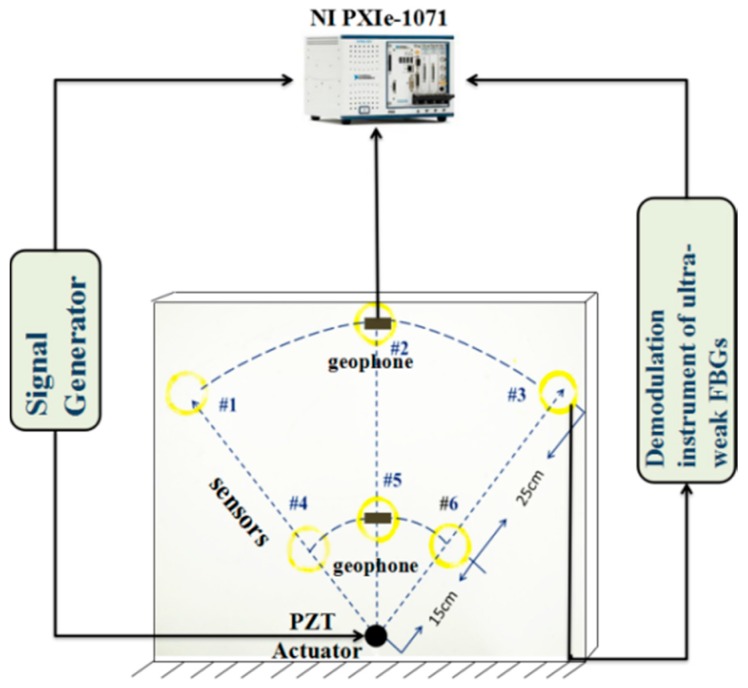
Configuration of the experimental sensor testing system showing the arrangement of sensors #1, #2, #3, #4, #5, #6 and piezoelectric transducer (PZT) actuator on the particle board.

In order to investigate the performance of our scheme, six consecutive sensors were tested. The sensors, labeled as #1, #2, #3, #4, #5 and #6 respectively, were cemented on the particleboard in a spiral form of radius 30 mm, placed at locations forming a fan shape, as shown in Figure 3. Among them, sensors #1, #2 and #3 were located on the outer circle 40 cm away from the center, while sensors #4, #5 and #6 were located on the inner circle 15 cm away from the center, as shown in Table 1. The three corresponding sensor pairs aligned in the same radial direction are therefore #1 and #4, #2 and #5, #3 and #6.

**Figure 4:**
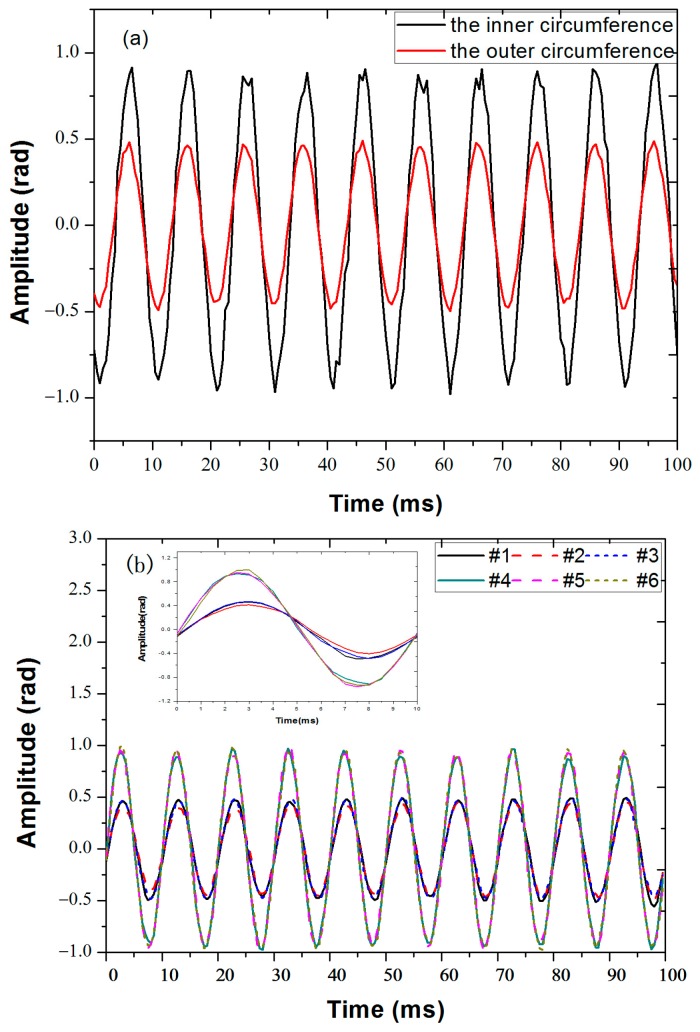
The time domain signals detected by (**a**) geophone and (**b**) the six fiber sensors at locations marked in Figure 3. The PZT source is driven by a sinusoidal voltage signal of 100 Hz with an amplitude of 1 V. Vertical axes have been normalized such that the average amplitude of the sensors at the inner radius is 1.

Under the same incident condition, the amplitude of the acoustic pressure signal arriving at sensors placed at the same distance away from the center should be the same, if edge reflections of the vibration signal are ignored. The acoustic intensity, corresponding to the pressure squared, will be approximately inversely proportional to the sensor distance from the vibration source. The inverse-distance relationship (rather than inverse-distance-squared relationship) is due to the fact that the acoustic signal is confined in a two-dimensional plank, rather than propagating in three-dimensional space. However, due to the fact that not all acoustic energy is confined in the plank, the inverse-distance relationship is only approximate. In addition, there is signal attenuation which is related to the frequency of the vibration signal and the internal structure of the particleboard [16]. In order to obtain more precisely the attenuation between signals at the two distances, a conventional geophone (SD-SN4) was placed on the inner and outer circles respectively [16]. When the function generator was set to output a sine-wave at 100 Hz with an amplitude of 1 V, the obtained results detected by the geophone at sensor locations #2 and #5 are given in Figure 4a. The measured amplitudes of the geophone signals in the inner and outer circle are A_i_ = 0.046 ± 0.03 rad, A_o_ = 0.023 ± 0.01 rad on average, thus the acoustic intensity ratio at the two radii from the center is [−20log_10_(A_o_/A_i_) = 6.04 dB]. Note the attenuation is greater than 4.26 dB, the ratio of the two radii [10log10(40/15) = 4.26 dB], indicating that in addition to the inverse-distance rate of decay, acoustic energy leakage away from the plank as well as absorption of the plank also contribute to signal attenuation. The signals collected by the six sensors are shown in Figure 4b, displaying near ideal sinusoidal shapes. As expected, the signals from the three sensors placed on the outer radius almost overlap with each other, with average amplitudes A_1_ = 0.478 ± 0.010 rad, A_2_ = 0.432 ± 0.019 rad, A_3_ = 0.479 ± 0.013 rad. Similarly, the signals from the three sensors placed on the inner radius also overlap well, with average amplitudes A_4_ = 0.916 ± 0.034 rad, A_5_ = 0.943 ± 0.025 rad, A_6_ = 0.948 ± 0. 030 rad. In the radial direction, the amplitudes of vibration signals collected by the two sensors (i.e., sensors #1 and #4, sensors #2 and #5, sensors #3 and #6) decrease with increasing distance from the source, as expected. The measured attenuation of the vibration propagating in the radial direction by the fiber sensors is 5.66 dB, 6.60 dB, 5.94 dB respectively. Comparing Figure 4a,b, signals measured by fiber sensors agree well with the signals measured by the geophone, and the attenuation ratios detected by the fiber sensors in the radial direction are closely matched to that measured by the geophone.

**Figure 5:**
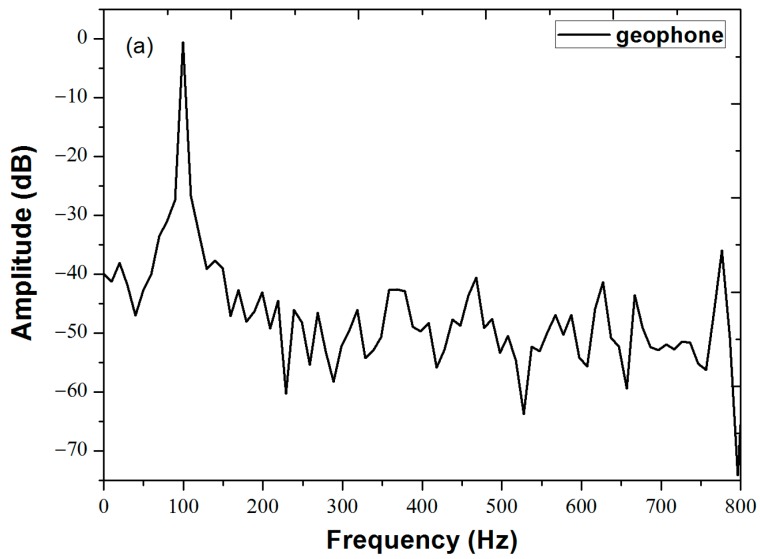

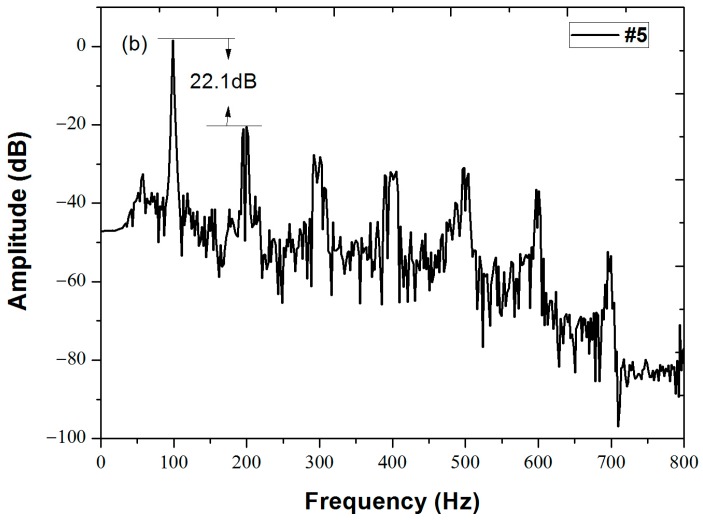
The frequency responses of vibration signals (obtained by fast Fourier transform (FFT) of the time domain signals) detected by (**a**) the geophone placed at the inner circumference, and by (**b**) fiber sensor #5.

The spectral analysis via fast Fourier transform (FFT) results of the signal detected by the geophone and the fiber sensor #5 are given in Figure 5a,b respectively. The vibration signals of sensor #5 show a primary spectral peak at 100 Hz, indicating that the demodulation algorithm is successful in extracting the frequency of the interference signals. Compared with the result shown in Figure 5a, the spectrum of the signal detected by sensor #5 contains harmonic peaks at 200 Hz, 300 Hz, 400 Hz, etc., which can be attributed to the asymmetry splitting ratio of the 3 × 3 coupler [11]. The 100 Hz peak is at least 22.1 dB higher than that of the higher harmonic peaks, which indicates that our system can properly demodulate the amplitude and frequency of the six vibration signals with a higher signal to noise ratio simultaneously.

**Figure 6:**
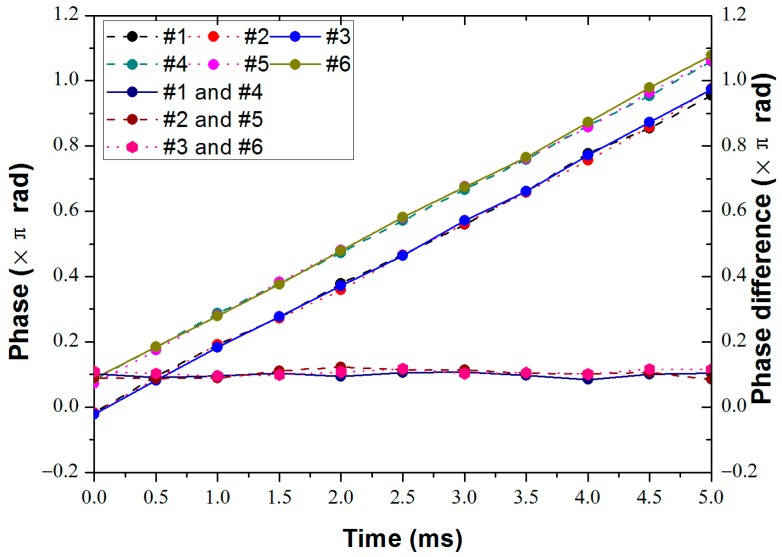
The phases of the vibration signal detected by the six sensors and the phase difference between sensors #1 and #4, sensors #2 and #5, sensors #3 and #6.

We can also see from Figure 4b and its enlarged view shown in the inset that there is a slight phase difference between the signals collected by the two sensors in the radial direction, caused by the finite propagating speed of the acoustic wave inside the particle board. The phases of the signals can be obtained through the demodulation algorithm, and the measured phases from 0 to 5 ms are shown in Figure 6; the phase differences between the two sensors in the radial directions are also shown in Figure 6. The phases of the signals at all sensors at 4 ms are shown in Table 1.

This shows that the phase of the vibration signals detected by three sensors in the circumferential direction are essentially the same, while there are constant phase differences between the sensor pairs in the radial direction. After averaging the experimental data, the phase difference between sensors #1 and #4 is *δ*_14_ = 0.099 ± 0.006π, that between sensors #2 and #5 is *δ*_25_ = 0.102 ± 0.010π, and that between sensors #3 and #6 is *δ*_36_ *=* 0.106 ± 0.007π. As the distance between inner and outer radii is *Δl* = 25 cm, the measured acoustic speed in the particle board at f = 100 Hz can be calculated as *v* = *fλ = fΔl(2π*/*δ)*, which gives 521 m/s, 490 m/s, 472 m/s, from the three radial pairs of sensors respectively. These values are in close agreement with the acoustic velocity of 500 m/s in lignocellulosic fibers (which is what most particle boards are made of) reported in the literature [17]. This indicates that our system can accurately extract the phases of the vibration signals from different locations simultaneously, which provides a feasible solution for vibration source positioning via detecting the phase of the signal.

**Figure 7:**
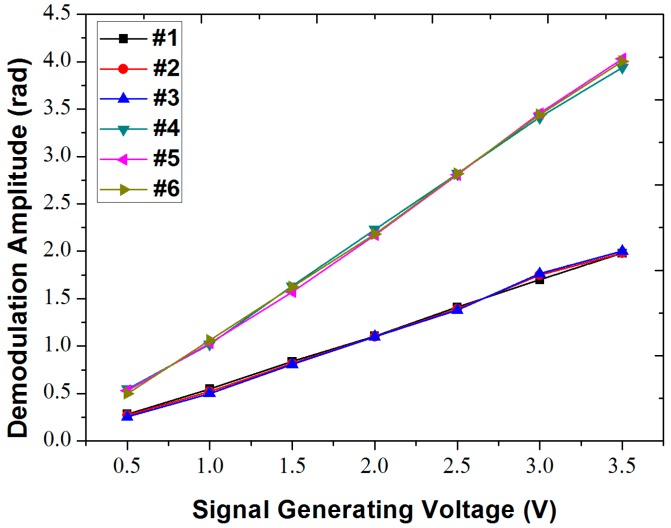
Demodulated amplitude information with different function generating voltages (0.5–3.5 V) at 100 Hz.

By increasing the voltage of the sine signal at the frequency of 100 Hz from 0.5 to 3.5 V with intervals of 0.5 V, a series of amplitude values were obtained. As can be seen from Figure 7 and Table 1, all six sensors exhibit a good linear relation with the driving voltage of the vibration signal. The signal amplitudes detected by the sensors in the same circumferential direction (i.e., sensors #1, #2 and #3, sensors #4, #5 and #6) are close in value and keep the same degree of linearity. In the radial direction (i.e., sensors #1 and #4), the signal amplitude detected by the two sensors maintains a constant attenuation coefficient. From the results, we can draw the conclusion that the system can extract the relative amplitudes of the vibration signals with high accuracy.

Next, we estimate the sensitivity of our sensors. Knowing the electrical power delivered to the PZT source and by estimating the efficiency of this power converted to the acoustic signal and coupled into the particle board, we can estimate the acoustic intensity (I_sound_) at the various sensing sites assuming the acoustic energy is radially spreading in a 2D plank and ignoring edge reflections. Then, using the relation I_sound_ = (P_rms_)^2^/*rv*, where P_rms_ is the root-mean squared pressure of the sound wave, *r* is the density of the particle board, and *v* is the sound velocity, we can calculate the pressure of the sound wave reaching the inner radius sites to be approximately 50 Pa/V of PZT drive voltage. The slope of the amplitude response of the inner-radius sensor shown in Figure 7 is ~1.2 rad/V, and therefore the sensitivity of our sensors is estimated to be ~0.024 rad/Pa. This value is similar to the value (0.038 rad/Pa) reported in [13], which has an effective sensing length of 5 m of optical fiber. In order to determine the dynamic range of the system, the background noise floor is obtained when the function generator is off, which is 1 × 10^−3^ rad on average, corresponding to an acoustic pressure of 0.04 Pa. The maximum pressure that can be measured exceeds what we can test using our PZT source. However, if we take the maximum unambiguous phase measurement to be 2π, then, the corresponding maximum pressure is 2.6 × 10^2^ Pa. Therefore, the dynamic range of the system is estimated to be ~38 dB.

**Figure 8:**
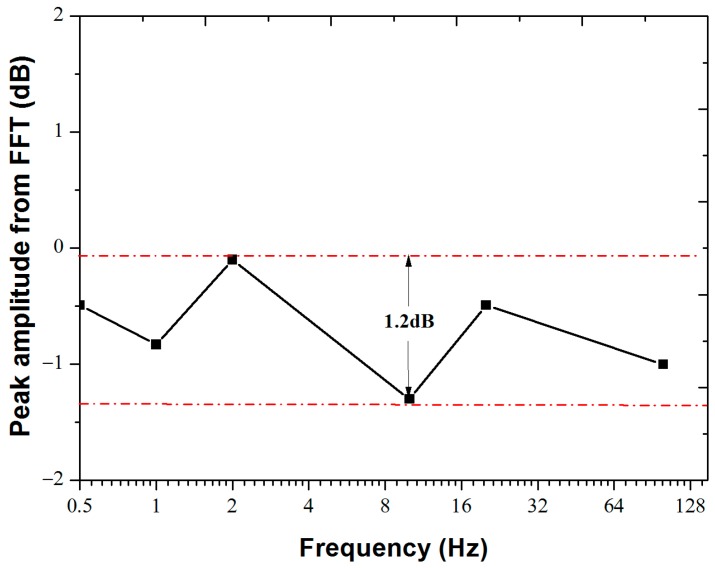
The frequency response of sensor #3 at 0.5 Hz, 1 Hz, 2 Hz, 10 Hz, 20 Hz, and 100 Hz with a driving PZT amplitude of 1 V.

To demonstrate the broadband capability of our system, we operated the system at 0.5 Hz, 1 Hz, 2 Hz, 10 Hz, 20 Hz, and 100 Hz, with a PZT driving voltage amplitude of 1 V for all cases (Figure 8). The average peak amplitudes of the demodulated signals were −0.49 dB at 0.5 Hz, −0.83 dB at 1 Hz, −0.10 dB at 2 Hz, −1.30 dB at 10 Hz, −0.49 dB at 20 Hz, and −1.0 dB at 100 Hz. The response flatness of less than 1.2 dB was obtained over 7.3 octaves of a frequency range from 0.5 Hz to 100 Hz.

We note here that Figure 8 presents the frequency response of the entire system, which includes frequency responses of the PZT source, of the particle board, as well as that of the fiber-optic sensing system. Given that the size of the particle board is much smaller than the acoustic wavelengths of the signal in the 0.5–100 Hz frequency range, and given that the PZT source data sheet does not show any resonant frequency response in the 0.5–100 Hz frequency range, we conclude that both the particle board and the PZT source have a relatively flat frequency response in the test range. Therefore, the flatness of the frequency response shown in Figure 8 is representative of the frequency response of the fiber-optic sensing system. The flat frequency response and the low noise over 7.3 octaves of the challenging low frequency range of 0.5–100 Hz indicate that our system is promising for submarine hydrophone applications [18].

## 4. Conclusions

We demonstrated location-resolved low-frequency vibration sensing based on Fizeau interference in a fiber-optic system consisting of 500 identical uwFBGs equally spaced by 5 m, for a total sensing length of 2.5 km. We have conducted detailed experimental investigation on the performance of six consecutive sensors in this array and demonstrated that the system is capable of demodulating the amplitude, phase, and frequency of the vibration signals for all sensors simultaneously. Using an external geophone sensor, we have independently verified the accuracy and linearity of our sensor system’s amplitude and frequency responses. The SNR of 22.1 dB is obtained at 100 Hz, and the estimated sensitivity of our system is 0.024 rad/Pa at 100 Hz; similar to other fiber-optic vibration sensing systems, the dynamic range of the system is estimated to be ~38 dB. The measured phase differences of signals at different distances away from the source predict an average acoustic speed of ~490 m/s at 100 Hz in a particle board, consistent with the known velocity of sound waves in particle boards consisting mainly of lignocellulosic fibers. Hence, the accuracy of the phase measurement is also indirectly verified. Finally, we demonstrate the frequency response of the system with a flatness of <1.2 dB over 7.3 octaves of the frequency range from 0.5 Hz to 100 Hz. Compared to the prior state-of-the-art demonstration using a similar method [12], our system has increased the sensing range from 1 km to 2.5 km, and increased the frequency range from 0.4 octaves to 7.6 octaves, in addition to achieving sensing in the very challenging low frequency range of 0.5–100 Hz.

## Figures and Tables

**Table 1:** The distance from the vibration source of all six sensors; the phase of the vibration signal detected by sensors at 4 ms, and amplitude response of all six sensors.

Sensor Number	#1	#2	#3	#4	#5	#6
Distance from vibration source (cm)	40 ± 1	40 ± 1	40 ± 1	15 ± 1	15 ± 1	15 ± 1
Phase of the vibration signal detected by sensors at 4 ms (π rad)	0.777 ± 0.001	0.756 ± 0.001	0.772 ± 0.001	0.862 ± 0.001	0.873 ± 0.001	0.873 ± 0.001
Sensor amplitude response (rad/V)	0.57 ± 0.01	0.58 ± 0.01	0.59 ± 0.01	1.18 ± 0.01	1.18 ± 0.01	1.15 ± 0.01
